# Tear and Saliva Metabolomics in Evaporative Dry Eye Disease in Females

**DOI:** 10.3390/metabo13111125

**Published:** 2023-11-02

**Authors:** Fredrik A. Fineide, Behzod Tashbayev, Katja B. P. Elgstøen, Elise M. Sandås, Helge Rootwelt, Håvard Hynne, Xiangjun Chen, Sten Ræder, Jelle Vehof, Darlene Dartt, Janicke L. Jensen, Tor P. Utheim

**Affiliations:** 1 The Norwegian Dry Eye Clinic, 0366 Oslo, Norway; 2 Department of Medical Biochemistry, Oslo University Hospital, 0450 Oslo, Norway; 3 Department of Computer Science, Oslo Metropolitan University, 0130 Oslo, Norway; 4 Department of Oral Surgery and Oral Medicine, Faculty of Dentistry, University of Oslo, 0313 Oslo, Norway; 5 Department of Ophthalmology, Drammen Hospital Trust, 3004 Drammen, Norway; 6 Department of Ophthalmology, Sørlandet Hospital Trust, 4838 Arendal, Norway; 7 Department of Ophthalmology, Oslo University Hospital, 0450 Oslo, Norway; 8 Departments of Ophthalmology and Epidemiology, University Medical Center Groningen, 9713 Groningen, The Netherlands; 9 Schepens Eye Research Institute/Massachusetts Eye and Ear, Department of Ophthalmology, Harvard Medical School, 20 Staniford St., Boston, MA 02114, USA; 10 Department of Oral Biology, Faculty of Dentistry, University of Oslo, 0313 Oslo, Norway

**Keywords:** dry eye disease, xerostomia, metabolomics

## Abstract

Accurate diagnosis of dry eye disease (DED) is challenging, and even today there is no gold standard biomarker of DED. Hypothesis-free global metabolomic studies of tears from DED patients have great potential to discover metabolites and pathways affected in the pathophysiology of DED, and to identify possible future biomarkers. These metabolites and biomarkers could be important for diagnosing and monitoring disease as well as for new therapeutic targets and strategies. As DED is associated with dry mouth, this study aimed to perform metabolomic analyses of tears and saliva from patients with decreased tear film break-up time but normal Schirmer test, and age-matched controls with both tear production and stability within physiological range. We applied strict inclusion criteria to reduce sampling bias in the metabolomic analyses and selected only age-matched females with Schirmer test values between 10–15 mm/5 min. The tear film analysis arm included 19 patients (with tear film break-up time 0–5 s) and 12 controls (with tear film break-up time 10–30 s), while the salivary analysis arm consisted of a subset which included 18 patients and six controls. Metabolomic analyses were performed using liquid chromatography and high-resolution mass spectrometry. Analyses using a global database search detected a total of 56 metabolites in tear samples that were significantly different between the groups. Of these, several have known associations with DED. These metabolites are present in meibum and have anti-oxidative characteristics or associations with the ocular microbiome, and altered concentrations suggest that they may play a significant role in DED associated with decreased tear film stability. In saliva, hypotaurine levels were lower among patients with tear film instability. In this pilot study, we found different levels of several metabolites in patients with decreased tear film break-up time that may have associations with DED. Future studies are required to replicate our findings and clarify the exact roles of these metabolites.

## 1. Introduction

In the last decade, dry eye disease (DED) has received increasing attention, as it is one of the most common diseases in ophthalmology, a curative treatment is currently not available, and symptomatic treatment is often unsatisfactory. Dry eye disease is defined as “… a multifactorial disease of the ocular surface characterized by a loss of homeostasis of the tear film, and accompanied by ocular symptoms, in which tear film instability and hyperosmolarity, ocular surface inflammation and damage, and neurosensory abnormalities play etiological roles” [1]. Screen use is linked to the increased prevalence of DED [2]. As we live in an era of increasing digitalization, this may accelerate disease development, making research on DED increasingly more relevant. Moreover, part of this multifactorial genesis might stem from ocular dysbiosis [3]. Previous techniques have yielded limited results in mapping the ocular microbiome due to shortcomings of culture-based approaches [4]. With the advent of next-generation sequencing and metagenomics, novel information regarding the ocular microbiome is being disclosed [5]. This highly prevalent disease has a high socioeconomic burden as well as a significant negative effect on patient quality of life [6,7,8,9,10]. Traditionally, DED is diagnosed using questionnaires and a battery of clinical tests such as tear film break-up time (TFBUT), ocular surface staining (OSS), Schirmer test, and evaluation of meibomian gland functionality [11]. Even though there are multiple available diagnostic tests employed in clinical practice today, a single diagnostic test or combination of methods with sufficiently high sensitivity and specificity is not available.

Some patients with DED also suffer from a feeling of dryness in the oral cavity, known as xerostomia [12]. Decreased tear film and salivary function, with resultant dry eyes and mouth, is referred to as “sicca complex” or “sicca syndrome” [12].

Metabolomics is a relatively new research discipline that aims to identify a wide range of metabolites in body fluids and tissues. As of today, application of metabolomics in DED research is limited. There are few published reports identifying possible biomarkers of DED [13,14,15,16]. However, metabolomics is a promising and powerful tool for multidimensional analysis of the metabolic status of tear fluid and saliva [17]. This tool may help us gain better understanding of the pathophysiology of DED and xerostomia. Metabolomics may also aid in identifying future biomarkers of DED and xerostomia that can be used in early diagnosis and disease prevention as well as in monitoring of disease progression and treatment responses. Moreover, metabolomics may identify new therapeutic targets and novel treatment opportunities. In this pilot study, our objective was to identify differences in the metabolomic profile of tears and saliva among dry eye patients with decreased tear film stability and normal Schirmer test values and controls without pathological Schirmer test or TFBUT.

## 2. Materials and Methods

### 2.1. Patient Selection

**Figure 1:**
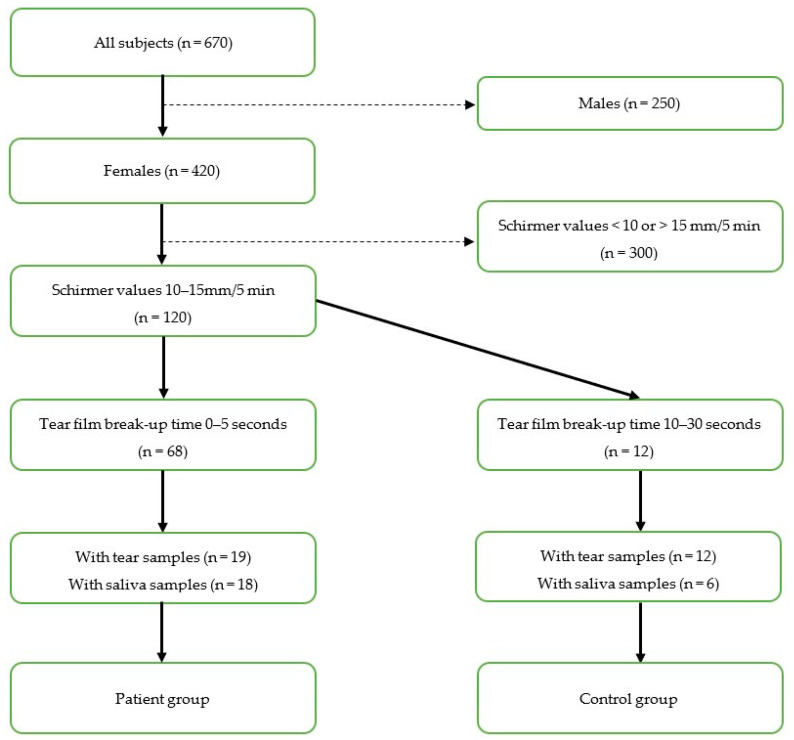
Flow diagram of selection process based on inclusion criteria.

This study is a part of a larger research project that was performed in collaboration between the Faculty of Dentistry, University of Oslo; the Norwegian Dry Eye Clinic; and the Department of Medical Biochemistry, Oslo University Hospital. The Norwegian Regional Committee for Medical and Health Research Ethics approved the study protocol (REK 2017/99), and the study was performed in compliance with the tenets of the Declaration of Helsinki. Patients visiting the Norwegian Dry Eye Clinic between March 2017 and February 2020 were included in the research project. Written informed consent was obtained from all subjects prior to participation. The study involved extensive evaluation of the ocular surface to characterize DED, tear sample collection with Schirmer test strips, and evaluation of oral dryness and saliva sample collection. To reduce noise in the interpretation of the analytical results, we made the groups as homogeneous as possible with respect to possible interfering factors. Therefore, we matched the age in the groups. Moreover, we selected only females with unanesthetized Schirmer values between 10–15 mm/5 min in at least one eye. Patients were assigned to the low TFBUT group if TFBUT was 0–5 s, hereby referred to as the patient group. Subjects with a TFBUT of 10–30 s were assigned to the high TFBUT group, hereafter referred to as the control group. Of a total of 670 subjects in the Norwegian Dry Eye Clinic, 420 were females, of whom 120 had Schirmer values between 10 and 15 mm/5 min. Sixty-eight patients had TFBUT 0–5 s. Of these 68 patients, we had access to tear and saliva samples from 19 and 18 patients, respectively. In the control group, we had access to tear samples from 12 subjects, and saliva samples from six subjects. The final number of participants in the tear film analysis arm and salivary analysis arm thus were 19 patients and 12 controls and 18 patients and six controls, respectively. The selection process is shown in Figure 1. Tear and saliva samples from the included subjects were subjected to metabolomic analyses. Comparative characteristics of the patient and control groups are shown in Table 1.

### 2.2. Tear Sample Collection

Tear samples were collected with standardized Schirmer test strips (HAAG-STREIT, Essex, UK). At the end of the Schirmer test, each strip was transferred to an Eppendorf tube containing 500 µL of 0.1 µm-filtered phosphate-buffered saline (PBS) pH 7.4 (Gibco, Thermo Fisher Scientific, Oslo, Norway), and then stored at −80 °C until metabolomic analyses. This method of sampling is the method of choice for proteomics, but not for metabolomic analysis.

### 2.3. Saliva Sample Collection

Stimulated whole saliva (SWS) was collected according to a standardized predefined protocol. The saliva was collected having the participant chewing on a paraffin pellet (Ivoclar Viavadent, Shaan, Lichenstein). The paraffin pellets were chewed for about 30 s, or until soft. The participants were then asked to swallow all the saliva in the mouth, and to continue chewing. During the collection the patients were asked to spit regularly in a plastic cup, and to avoid talking. All saliva samples were chilled on ice during collection and sampling. The samples were weighed to the closest decigram and aliquoted to 0.5 mL before being transferred to Eppendorf tubes and stored at −80 °C.

### 2.4. Materials

LC-MS chemicals and solvents: All water used was of type 1 (resistivity >18 MΩ-cm at 25 °C), obtained from Milli-Q ultrapure water purification system (Merck Millipore, Darmstadt, Germany). Methanol (LC-MS grade) was obtained from Rathburn Chemicals (Walkerburn, Scotland). Formic acid, LC-MS grade was obtained from Thermo Fisher Scientific, Waltham, MA, USA.

### 2.5. Sample Preparation

All samples were stored at −80 °C, thawed at room temperature, and mixed prior to analyses.

Tear samples: Tear samples were thermomixed for 45 min at 45 °C and 700 rpm. Schirmer strips were completely soaked in the solvent and vortexed. An aliquot of 50 µL from each tear sample was transferred to a micro tube and mixed to a pooled quality control (PQC) sample.

Saliva samples: An aliquot of 100 µL of each saliva sample was transferred to a micro tube with 300 µL methanol (4 °C). The samples were centrifuged for 10 min at 4 °C and 14,000× *g*. An aliquot of 50 µL from each saliva sample was transferred to a micro tube and mixed to a PQC sample.

The tear samples, saliva samples, and PQC samples were transferred to separate high-performance liquid chromatography (HPLC)-vials, and 2 µL was injected for analysis.

Water was used as a blank sample and prepared in the same way as the samples prior to analysis.

### 2.6. Metabolomics Analyses/LC-MS Analysis

Tear and saliva samples were analyzed in random order using the LC-mass spectrometry (MS) metabolomics method described in [18], in separate runs. The blank sample was analyzed at the beginning of the sample sequence. The PQC sample was injected between every fifth sample injection. Additionally, the PQC sample was analyzed using data-dependent MSMS (ddMS^2^, top 5) scan mode (Settings: Resolution (at *m*/*z* 200): 17,500. Automatic Gain Control target value: 500,000 ion counts. Maximum injection time: 100 ms. Loop count: 5. Isolation window: *m*/*z* 1.0. Fixed first mass: *m*/*z* 50.0. Stepped (N) CE: 20, 50, 80), injected both at the beginning and the end of the sample sequence. The samples were analyzed in two separate injections with both positive and negative ionization mode in the electrospray ionization source.

Software used for data acquisition were Xcalibur v. 4.2.47, Tune 2.11, and SII for Xcalibur 1.5, all from Thermo Fisher Scientific.

### 2.7. Statistics and Identification

Statistical software: Compound Discoverer 3.1 (from Thermo Scientific) was used for data processing and statistical analyses, using the workflow template: “Untargeted Metabolomics with Statistics Detect Unknowns with ID using Online Databases and mLogic”. The statistical analyses used were principal component analysis (PCA), Shapiro–Wilk test to evaluate distribution of the data, and Mann–Whitney U-test to calculate the *p*-value of the differences between the groups. All results including two-tailed Student’s *t*-test and correction for multiple testing with the Benjamini–Hochberg method are included in Supplementary Table S1. Trend charts for each feature were created using the chromatographic peak area to compare the differences between all samples (trendline plot) and between patient and control groups (box–whisker plot).

The identification process in the workflow gave tentative identification of features generated by the LC-MS analysis by using the following databases:

- ChemSpider (http://www.chemspider.com/ (accessed on 12 July 2020)) database was used to search FullMS scans by using the molecular weight or predicted formulas when available.
- mzCloud: (https://www.mzcloud.org/ (accessed on 12 July 2020)) database was used to search MSMS scans by using the fragmentation pattern.

## 3. Results

The characteristics of patients and controls are given in Table 1 and are in line with the selection criteria.

### 3.1. Tear Metabolomics

**Figure 2:**
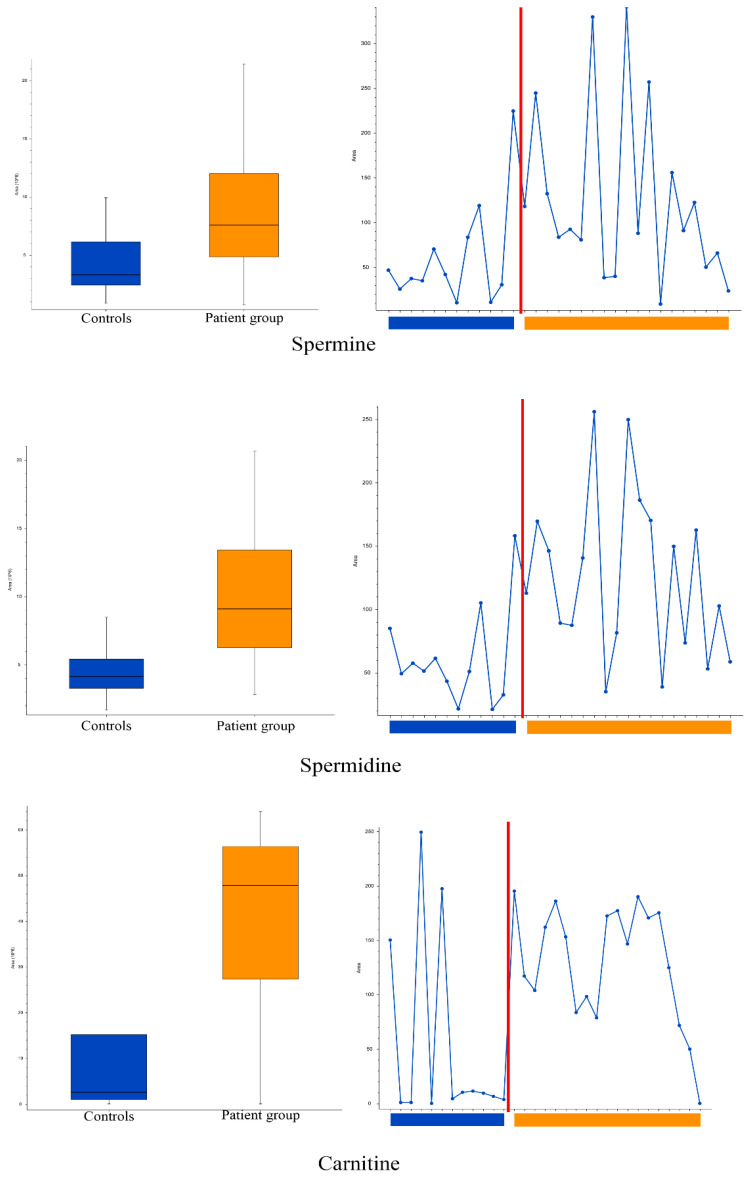
Box, whisker and trendline plots: comparison of spermine (**top**), spermidine (**middle**) and carnitine (**bottom**) levels in the tears of patient and control groups. Boxplot parameters: interquartile range = quartile 3 (Q3) − quartile 1 (Q1); upper whisker = Q3 + IQR × 1.5; lower whisker = Q1 − IQR × 1.5. Boxplot parameters from top: upper whisker; Q3; median; Q1; lower whisker.

**Figure 3:**
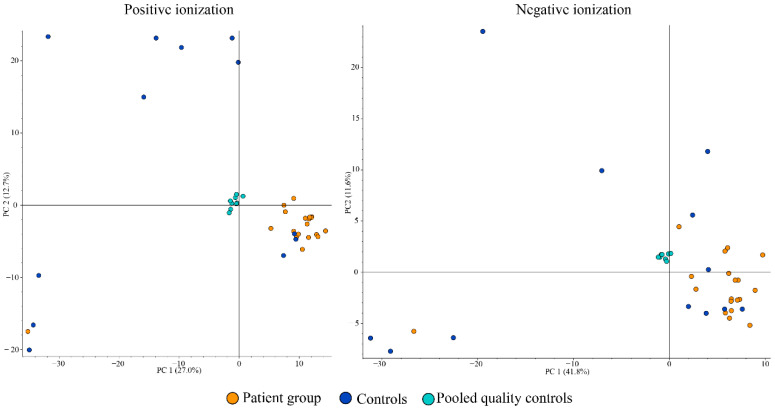
Positive ionization principal components analysis (PCA) (**left**) and negative ionization PCA (**right**) of tear samples.

The global metabolomic analyses of tear samples detected a total of 56 features with significantly (*p* < 0.05) different peak areas (ratio of <0.5 or >2) between the patient and control groups. This is the standard approach for global metabolomics analysis for identifying potential biomarkers separating the two groups. Level of confidence of identification are outlined in Table 2 and metabolites with tentative identification are listed in Table 3 and Table 4. The complete list of the 56 features can be found in Table S1 in the Supplementary Materials. Based on database searches, we tentatively identified many potential metabolites that are not yet known to have an association with DED as well as metabolites with known DED association, namely carnitine, spermine, and spermidine (Figure 2). The patient group had almost 19 times higher amount of carnitine compared to the control group (group areas of controls vs. patients: 2.5 × 10^6^ vs. 47.9 × 10^6^, *p* = 0.02). The patient group also had higher amount of spermine (group areas of controls vs. patients: 3.3 × 10^6^ vs. 7.6 × 10^6^, *p* = 0.025) and spermidine (group areas of controls vs. patients: 4.1 × 10^6^ vs. 9.1 × 10^6^, *p* = 0.003). Principal component analysis (PCA) was performed and is demonstrated in Figure 3, in which clustering of the patient group in both positive and negative ionization mode can be observed, with some exceptions.

Moreover, the three features with suggested metabolite identity that had the highest ratios in the patient group and significant *p*-values were glycyltyrosine (301:1); (2S)-2-Piperazinecarboxylic acid (15.9:1), and O-(2-Aminoethyl)serine (13.3:1). The three metabolites with the lowest ratios in the patient group were 3,4,5-trimethoxycinnamic acid (0.007), Panthenol (0.044), and Resorcinol (0.091). These six tentative metabolites and their functions are summarized in Table 5.

### 3.2. Saliva Metabolomics

**Figure 4:**
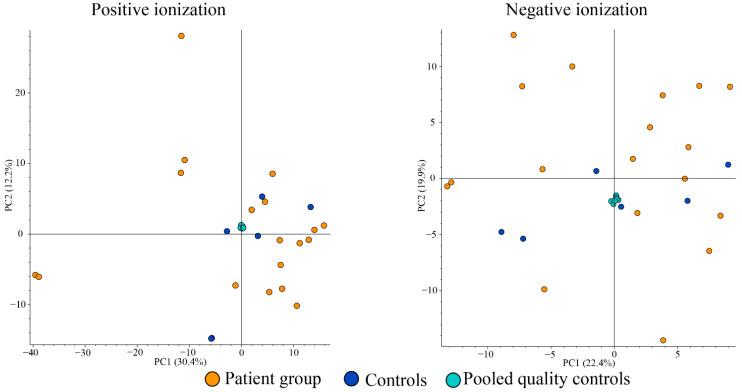
Principal components analysis plots from positive (**left**) and negative ionizations (**right**) of saliva samples.

Comparison of saliva metabolites with PCA demonstrated no apparent clustering of or separation between the patient or control groups (Figure 4).

**Figure 5:**
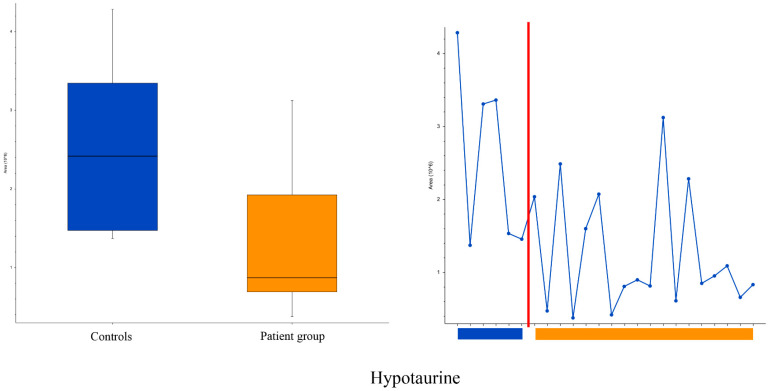
Box, whisker (**left**) and trendline (**right**) plots of hypotaurine levels in saliva in evaporative dry eye patients and controls. Controls to the left and patients to the right of the red line. Boxplot parameters: interquartile range = quartile 3 (Q3) − quartile 1 (Q1); upper whisker = Q3 + IQR × 1.5; lower whisker = Q1 − IQR × 1.5. Boxplot parameters from top: upper whisker; Q3; median; Q1; lower whisker.

In positive ionization, we observed a total of 499 peaks; two of the peaks (fluorescein and hypotaurine) were significantly different between the groups, with fold changes of <0.5 or >2. Fluorescein was excluded, as it originates from the ophthalmic examination (TFBUT). Hypotaurine levels were lower in the patient group (2.4 × 10^6^ vs. 0.9 × 10^6^, *p* = 0.015), as illustrated in Figure 5, predominantly due to very low values in a subgroup of the patients. In negative ionization, a total of 170 peaks were observed, but none of them was significantly different (*p* > 0.05) between the groups.

## 4. Discussion

This study showed significantly different levels of several tear metabolites between patients with decreased TFBUT and controls with normal TFBUT and Schirmer values. The tear metabolomic study produced a large number of tentative metabolites (Table 3 and Table 4), of which some are known to play a role in DED while others are not. Among salivary metabolites, only hypotaurine demonstrated a between-group difference, with a lower amount detected in patients with low TFBUT, particularly evident in a subgroup with very low values as demonstrated in the trendline plots. This may reflect the differences expected to be present in patients with DED due to various etiologies, including the biochemical pathways and molecular networks that are predominantly affected, and the stage of the disease at the time of analysis.

In our analyses, the patient group had 19 times higher tear levels (defined by area under the curve) of carnitine than the control group. There is general agreement that carnitine plays an important role in reducing oxidative stress [32]. The pathophysiology of evaporative DED is complex, but a general mechanism can be summarized as follows: (1) lack of adequate meibum leads to (2) unstable tear film that evaporates easily, causing (3) hyperosmolarity in the remaining tear fluid, which in turn instigates (4) oxidative stress potentially leading to (5) oxidative damage of the ocular surface. Carnitine is known to have a protective effect against oxidative stress and inflammatory responses in human corneal epithelial cells [33].

Hua et al. tested whether L-carnitine had a protective effect against oxidative damage in human corneal epithelial cells (HCECs) induced by hyperosmolarity [33]. The cells were cultured in a hyperosmolar medium with 450 mOsm with and without L-carnitine for up to 48 h. The L-carnitine supplemented medium showed less production of reactive oxygen species. Moreover, L-carnitine decreased other oxidative markers induced by hyperosmolarity, such as cytotoxic membrane lipid peroxidation levels and protein production of heme oxygenase. In a study by Deng et al. [34], L-carnitine caused reduced production and activity of matrix metalloproteinases (MMPs) in primary human corneal epithelial cells exposed to hyperosmotic stress. Chen and co-authors [35] evaluated the efficacy of L-carnitine, betaine, and erythriol on prevention and treatment of DED in a murine model. Compared to phosphate-buffered saline (PBS), the combination of L-carnitine, betaine, and erythriol significantly decreased corneal staining and expression of Tumor Necrosis Factor-α (TNF-α) and Interleukin-17 (IL-17). More specifically, application of L-carnitine containing drops for 14 days resulted in a significant reduction in corneal staining, the number of TUNEL-positive cells, and the expression of TNF-α and interleukins (IL-17, IL-6, or IL-1β), as well as significantly increasing the number of goblet cells. These findings imply that carnitine may have an anti-inflammatory role.

To date, few metabolites have been shown to protect against hyperosmolarity, named osmoprotectants. Examples include L-carnitine, erythriol, and betaine, which are occasionally used in formulations for treatment of DED (Optive Plus^TM^ and Isomar Eyes Plus). Several randomized studies have reported advantages of adding osmoprotectants in artificial tear substitutes used in DED treatment. A randomized controlled trial included 47 DED patients treated with either eye drops containing carboxyl methylcellulose plus osmoprotectants (erythriol and L-carnitine) or hyaluronate-based products without osmoprotectants. The study demonstrated that eyedrops containing osmoprotectants had greater effect on the reduction of conjunctival staining at one month. Nebbioso et al. conducted a randomized controlled trial comparing the effect of eye drops containing carnitine, taurine, eledoisin, and sodium hyaluronate (Carnidrop Plus^®^) to controls treated with 0.9% saline solution eye drops in patients diagnosed with iatrogenic DED caused by benzalkonium chloride (BAK) containing glaucoma eye drops [36]. Patients in the treatment group had significant improvement of OSDI, Schirmer test, TFBUT, and ocular protection index after 15 days (all *p* < 0.0001). The control group treated with placebo drops, on the other hand, showed no significant improvement. A randomized, single-blinded study by Evangelista et al. compared treatment effects of three lubricant eye drop solutions in DED [37]. The study included 40 patients where three groups received either Carnidrop (L-carnitine titer 1%), Optive (L-carnitine titer = 0.258%), or BluSal (a physiological solution with no L-carnitine). The Carnidrop group demonstrated greater ability in increasing TFBUT over a one-hour period than the groups treated with lower concentration or no carnitine. Higher concentration of carnitine may result in greater improvement of tear film stability. Taken together, these reports imply that carnitine has a protective role against the biochemical and mechanical insults in DED. Our finding of 19-fold higher carnitine levels among patients with tear film instability thus indicate that this increase might be a physiological, compensatory response to reduce the damages of oxidative stress and inflammation in DED.

In our study, we also observed higher concentrations of spermine and spermidine in the patient group. These polyamines are hypothesized to have a protective effect against damage caused by oxidative stress [38]. Rider et al. tested this hypothesis in mouse fibroblast cells exposed to hydrogen peroxide [39]. The authors concluded that both spermine and spermidine can protect the cells from oxidative stress related damage. Interestingly, Jiang et al. found decreased levels of spermine in reflex tears of DED patients when compared to healthy controls, possibly due to dilution or inhibition of histidine and glutathione metabolism [40]. The other members of the histidine and glutathione metabolic pathway (urocanic acid, L-glutamic acid, pyroglutamic acid, and oxidized glutathione) were not identified in our study. Hence, the state of this metabolic pathway among our subjects is unknown.

Furthermore, we observed that the patient group had abnormally high ratios of some metabolites tentatively identified as glycyltyrosine (301:1), (2S)-2-Piperazinecarboxylic acid (15.9:1), and O-(2-Aminoethyl) serine (13.3:1). Glycyltyrosine is a dipeptide and secondary metabolite possibly associated with ingestion of certain meats and colorectal cancer that may serve as a signaling molecule [19,20]. Its possible role in DED is unknown. The compound C_5_H_10_N_2_O_2_ may represent (2S)-2-piperazinecarboxylic acid, aminoproline, cucurbitine, or 5-imino-L-norvaline. None of these has any known associations with DED; however, aminoproline, also known as 1-aminopyrrolidine-2-carboxylic acid, is involved in the proline pathway [21]. Proline is of importance to transcription factors, gene expression and synthesis of collagen, ornithine, glutamate, arginine, and polyamines [41]. Moreover, it is a metabolite found in *Escherichia coli* [42]. Increased expression of the proline pathway was reported by both Jiang et al. and Chen et al. among patients with DED compared to controls [40,43]. The molecular feature C_5_H_12_N_2_O_3_ has been tentatively identified as either O-(2-aminoethyl) serine or N(5)-hydroxyornithine. The former is an alpha amino acid and antimetabolic antibiotic from *Streptomyces reseoviridofuscus* [23]. The latter is involved in siderophore synthesis for both *Pseudomonas aeruginosa* and *Aspergillus fumigatus*, promoting biofilm formation and virulence [24,25]. *Pseudomonas* species have been found as both part of the healthy ocular flora as well as being upregulated in DED and meibomian gland dysfunction (MGD) [44,45].

Similarly, there are some tentatively identified metabolites with substantially lower ratios in the patient group. These include 3,4,5-trimethoxycinnamic acid (0.007), panthenol (0.044), resorcinol (0.091), diethanolamine (0.124); 1-deoxynojirimycin (0.133), oleamide (0.226) and pyridoxal (0.389). Importantly, at this point, we do not know the exact identity of most of the metabolites or whether they play a role in the pathogenesis of or as biomarkers for DED or not.

3,4,5-trimethoxycinnamic acid might be an allergen and an inhibitor of TNF-α-mediated cytokine release [26,27]. Pantothenic acid, or vitamin B5, is the carboxylic equivalent of panthenol, essential for the synthesis of coenzyme A and thus epithelial protein metabolism, skin regeneration, and wound healing [28,29]. Resorcinol is not a metabolite found in bacteria or humans; rather, it is an antiseptic that tends to be used in topical pharmaceutical products with keratolytic activity [30,31]. The role of these metabolites in DED needs both confirmation and further research.

The molecular feature C_4_H_11_NO_2_ may represent diethanolamine or aminoethyl propanediol. Both are used in the production of cosmetics [46,47], and both are possible xenobiotics [48,49]. The former has been associated with *Escherichia coli* [48], while the latter with the baker’s yeast *Saccharomyces cerevisiae* [49].

Four possibilities arise with the molecular feature C_6_H_13_NO_4_. First, bicine is a buffering agent with no known associations to DED or any metabolic pathways of clinical relevance. Second, 1-deoxynojirimycin is an iminosugar found in mulberry leaves and dayflower (*Commelina communis*) as well as some strains of *Bacillus* and *Streptomyces* [50]. It has several interesting features such as anti-hypertriglyceridemia, antiviral, and antihyperglycemic effects. Third, perosamine is a dideoxy sugar found in the O-antigens of *Vibrio cholerae* and *Escherichia coli* serotype O157:H7 [51,52]. Finally, migalastat is a pharmacological chaperone to the enzyme deficient in Fabry disease, alpha-glactosidase [53].

Oleamide is the fatty acid amide derived from oleic acid and is endogenously produced in humans as a sleep-inducing agent and a neurotransmitter [54]. Nichols et al. proposed oleamide as a lipid of meibomian gland origin with an important role in tear film lipid stabilization [55]. This role was later questioned, as the reported spectra were similar to those seen when plasticware was exposed to solvents, thus Butovich concluded the findings reported by Nichols et al. probably were the result of contamination [54]. In our current analysis, we not only detected oleamide but found significant differences in oleamide concentration between the two included groups. Our findings are contrary to those reported by Jiang et al., who reported an increased concentration of oleamide among DED patients [40].

Pyridoxal is one of three pyridine derivatives of vitamin B6, whose active form is pyridoxal 5′-phosphate [56]. Pyridoxal 5′-phosphate serves as a cofactor for synthesis of amino acids and neurotransmitters as well as scavenging reactive oxygen species. Moreover, it must be dephosphorylated to pyridoxal in order to cross cell membranes, leave plasma, and enter target tissues, and its plasma concentration is inversely correlated to inflammatory markers.

The ocular microbiome is a complex mixture of the microorganisms inhabiting the facial skin, eyelids, conjunctiva, and cornea [57]. Dysregulation of several pathological and opportunistic bacterial species have been found in association with DED, MGD, and demodex blepharitis with the common finding of dysbiosis among patients. Blockage of the meibomian glands may increase the secretion of bacterial toxins causing tear film instability and inflammation [58]. Until recently, the charting of the ocular microbiome has been dependent on the ability to culture the microorganisms present on the ocular surface [4]. This hindrance is now being surpassed with next-generation sequencing and metagenomics, leading to the detection of potential eye community state types, quantifying different ocular bacterial profiles as well as the detection of commensal and pathogenic microbes not detected by standard culture-based approaches [5,59]. Interestingly, several of the potential metabolites identified (Table 5) may be xenobiotics of bacterial origin. This finding corroborates the importance of bacterial dysregulation in DED and tear film instability.

Some of the obtained tear metabolites (i.e., panthenol, oleamide, diethanolamine, or aminoethyl propanediol) may have originated from cosmetic product use, which may be avoided by DED patients and therefore downregulated in our study. Patients should be instructed not to use any cosmetic products prior to sample collection. However, traces of cosmetic products can remain for several days. It is therefore important to record type and possibly content of cosmetic products to rule out potential contamination of tear samples. Moreover, it may be that these cosmetic products lead to certain other subtypes of dry eye, explaining the differences between the groups in our study.

The patient group had lower levels of hypotaurine in saliva. Hypotaurine, usually an intermediate in the normal synthesis of taurine, is known to have an anti-oxidative effect [60,61,62]. It is unclear to us whether decreased levels of hypotaurine in saliva of the patient group may represent a decreased ability to withstand oxidative stress. Other differences in salivary metabolites were not found. A recent study compared the metabolomic profiles of healthy controls to that of patients with primary or secondary Sjögren’s syndrome [63]. Here, the authors identified several altered metabolites and presented alanine, isovaleric acid, and succinic acid as possible biomarkers for Sjögren’s syndrome. In the present study, focusing on dry eyes in general, but not on Sjögren’s syndrome in particular, great care was taken to identify strict inclusion criteria based on gender, age, normal secretion of tears, and access to samples of both tears and saliva. Patients and controls were separated based on TFBUT values only. In retrospect, the inclusion criteria could have been less rigid, focusing on tears alone.

A possible limitation of our pilot study is the recruitment of symptomatic individuals as controls. Although the control group had physiological values concerning Schirmer test and TFBUT, subjects in this group might have neuropathic dry eye. Another limitation is the tear sample collection method. Ideally, tear collection for the tear metabolomics analysis should be through microcapillaries [64]. However, this method is time-consuming and may not be possible to implement in a busy clinic. Another more practical method is tear collection by applying Schirmer strips and drying them in ambient temperature prior to preservation in an ultrafreezer. Although our tear collection method was not ideal for metabolomic analyses, the results demonstrate that it is possible to extract and quantify metabolites even in highly diluted medium such as a tear sample on a Schirmer strip preserved in 500 μL PBS. This sample collection method has previously been used successfully in our earlier studies investigating proteomics and cytokine levels in tear fluid [65]. Our demonstration that available high-quality biobank samples can be used for multiple purposes and multiomics, even when the sample preparation technique is not ideal for all analyses, is valuable.

Further strengths include access to tear samples from over 650 well characterized patients enabling us to make our groups internally homogeneous and distinct from each other. Moreover, we performed metabolomics analyses of tears and saliva simultaneously, and the quality of our metabolomics platform enabled us to detect a large range of metabolites, many of which differed in amounts between patients and controls.

## 5. Conclusions

Metabolomic studies of tears from DED patients have great potential for discovering metabolites and pathways of importance for understanding the pathophysiology of DED. The modality also enables the identification of possible future biomarkers for diagnostics and monitoring of disease progression, as well as pinpointing new possible therapeutic targets and strategies and the monitoring of treatments instituted. In the present work, we demonstrate that high-quality biobank samples can be used for multiomics, a valuable finding concerning future multiomics studies. Carnitine, spermine, aminoproline, oleamide, and spermidine in tears were found altered in this study and seem to have a significant involvement among DED patients with decreased TFBUT. Moreover, we detected several other metabolites with significant concentration differences between patients with tear film instability and controls. Larger studies confirming the metabolites detected in this work, their biochemical pathways, and possible mechanism of action in the pathogenesis of DED are warranted and are underway by our group.

## Figures and Tables

**Table 1:** Characteristics of selected subjects.

Characteristics	Patient Group (*n* = 19)	Control Group (*n* = 12)	*p*-Value
Age (years)	56.9 ± 17.3	54.4 ± 18.8	0.80
Female sex	100%	100%	
Schirmer value (mm/5 min)	12.0 ± 1.7	12.2 ± 1.6	0.74
TFBUT (seconds)	3.0 ± 1.2	14.6 ± 5.8	<0.0001
OSDI	35.8 ± 18.5	29.1 ± 20.2	0.37

Means ± standard deviations are presented. OSDI: Ocular Surface Disease Index; TFBUT: Tear film break-up time.

**Table 2:** Level of confidence of identification.

Level 1	Validated identification using in-house library of analytical standards (MS/MS spectrum and retention time match).
Level 2	Putative identification using online databases (MS/MS spectrum match).
Level 3	Putative identification supported by extra information.
Level 4	Tentative identification using online databases (chemical formula or molecular mass).
Level 5	Unique feature with *m*/*z* value and retention time.

**Table 3:** Tear samples. Positive ionization.

Increased in Patient Group								
Predicted Formula	Suggested Component Name(s)	Molecular Weight (g/mol)	Patient Group (Area 10^6^) (a.u.)	Control Group (Area 10^6^) (a.u.)	Retention Time (minutes)	Ratio	*p*-Value	Level of Confidence of Identification
C_10_H_26_N_4_	Spermine	202.22	7.6	3.3	1.426	2.292	0.025	2
C_7_H_19_N_3_	Spermidine	145.16	9.1	4.1	1.489	2.205	0.003	2
C_7_H_15_NO_3_	Carnitine	161.11	47.9	2.5	2.954	18.566	0.020	4
C_5_H_12_N_2_O_3_	O-(2-aminoethyl)serine; N(5)-hydroxyornithine	148.08	13.4	1.0	3.399	13.346	0.002	4
C_4_H_10_O_3_	Diethylene glycol; 1,2,3-butanetriol	106.06	518	140	4.120	3.689	0.001	4
C_5_H_10_N_2_O_2_	(2S)-2-piperazinecarboxylic acid; aminoproline; cucurbitine; 5-imino-L-norvaline	130.07	23.5	1.5	7.408	15.992	0.002	4
C_6_H_12_N_2_O_3_	Daminozide; dialanine; 4-acetoamido-2-aminobutanoic acid; methylglutamine	160.08	10.4	1.0	8.224	10.688	0.004	4
C_11_H_14_N_2_O_4_	Glycyltyrosine; 5-hydroxy-N-(4-hydroxyphenyl)-5-iminonorvaline	238.10	457.2	1.5	12.945	301.056	0.001	4
C_12_H_24_O_3_	12-hydroxylauric acid; 5-hydroxydodecanoic acid	216.17	21.9	7.4	14.277	2.948	0.016	4
**Decreased in Patient Group**								
C_2_H_7_NO	Ethanolamine; N,O-dimethylhydroxylamine	61.05	1.1	3.4	2.190	0.340	0.001	4
C_5_H_11_NO_4_	Triethanolamine; N,N-dihydroxy-L-valine	149.07	0.33	1.2	2.291	0.266	0.001	Triethanolamine: 2; N,N-dihydroxy-L-valine: 4
C_6_H_13_NO_4_	Bicine; 1-deoxynojirimycin; perosamine; migalastat	163.08	0.10	0.75	2.296	0.133	0.001	4
C_4_H_11_NO_2_	Diethanolamine; aminomethyl propanediol	105.08	0.48	3.8	2.405	0.124	0.007	4
C_8_H_9_NO_3_	Pyridoxal; methyl-4-aminosalicylate; 3,5,6-indolinetriol; orthocaine	167.05	3.9	10.0	3.255	0.389	0.001	4
C_9_H_19_NO_4_	Panthenol	205.13	0.44	10.1	10.625	0.044	0.012	2
C_6_H_6_O_2_	Hydroquinone; resorcinol; benzenediol	110.04	0.64	6.1	13.706	0.104	0.039	4
C_18_H_35_NO	Oleamide	281.27	14.1	62.5	19.060	0.226	0.001	2
C_22_H_44_O_3_	Hydroxydocosanoic acid	356.33	21.2	53.3	21.385	0.399	0.007	4

**Table 4:** Tear samples. Negative ionization.

Increased in Patient Group								
Predicted Formula	Suggested Component Name(s)	Molecular Weight (g/mol)	Patient Group (Area 10^6^) (a.u.)	Control Group (Area 10^6^) (a.u.)	Retention Time (minutes)	Ratio	*p*-Value	Level of Confidence of Identification
C_10_H_10_O_5_	3-(3-hydroxy-4-methoxyphenyl)-2-oxiranecarboxylic acid; 2,4-diacetylphloroglucinol; hydroxyferulic acid	210.05	13.7	3.2	12.631	4.263	0.001	4
C_25_H_22_O_9_	Silandrin	466.13	48.2	6.3	13.721	7.695	0.008	4
C_20_H_32_O_2_	Arachidonic acid; drosthanolone	304.24	4.0	1.9	20.442	2.102	0.039	4
C_22_H_32_O_2_	Retinyl acetate; docosahexanoic acid; methylprogesterone	328.24	2.0	0.39	21.014	5.128	0.012	4
**Decreased in Patient Group**								
C_9_H_19_NO_4_	Panthenol	205.13	0.22	4.2	10.603	0.053	0.020	2
C_12_H_14_O_5_	3,4,5-trimethoxycinnamic acid; 3-(6,7-dimethoxy-1,3-benzodioxol-5-yl)-2-propen-1-ol	238.08	0.03	3.6	13.183	0.007	0.003	4
C_6_H_6_O_2_	Resorcinol; cathecol	110.04	0.53	5.8	13.704	0.091	0.028	2
C_22_H_44_O_3_	Hydroxydocosanoic acid	356.33	14.5	34.2	21.381	0.424	0.011	4
C_22_H_42_O_4_	Adipid acid di(2-ethylhexyl) ester; docosanedioic acid	370.31	4.7	10.4	21.415	0.456	0.011	4

**Table 5:** The three most increased and decreased tentative metabolites and their possible functions.

Increased in Patient Group			
Predicted Formula	Suggested Component Name(s)	Function	Level of Confidence of Identification
C_11_H_14_N_2_O_4_	Glycyltyrosine; 5-hydroxy-N-(4-hydroxyphenyl)-5-iminonorvaline	Glycyltyrosine: belongs to peptides, might be associated with colorectal cancer [19]. Has been detected in poultry and pigs, might indicate human consumption of this meat [20]. Secondary metabolite and as such may serve a role in defense or signaling molecule [20]. 5-hydroxy-N-(4-hydroxyphenyl)-5-iminonorvaline: no relevant information.	4
C_5_H_10_N_2_O_2_	(2S)-2-piperazinecarboxylic acid; aminoproline; cucurbitine; 5-imino-L-norvaline	(2S)-2-piperazinecarboxylic acid: no relevant information. Aminoproline: also known as 1-aminopyrrolidine-2-carboxylic acid is a proline derivative [21]. Cucurbitine: exogenous alpha amino acid found in muskmelon, cucurbita seeds, and cucumber, might indicate consumption [22]. 5-imino-L-norvaline: no relevant information.	4
C_5_H_12_N_2_O_3_	O-(2-aminoethyl)serine; N(5)-hydroxyornithine	O-(2-aminoethyl)serine: is an alpha amino acid [23]. It is obtained from *Streptomyces reseoviridofuscus* and is an antimetabolic antibiotic. N(5)-hydroxyornithine: involved in siderophore synthesis in *Pseudomonas aeruginosa* and *Aspergillus fumigatus*, promoting virulence and biofilm formation [24,25].	4
**Decreased in Patient Group**			
C_12_H_14_O_5_	3,4,5-trimethoxycinnamic acid; 3-(6,7-dimethoxy-1,3-benzodioxol-5-yl)-2-propen-1-ol	3,4,5-trimethoxycinnamic acid: found in normal human urine and *Piper longum*, has a role as an allergen, and inhibits TNF-α induced cytokine expression [26,27]. 3-(6,7-dimethoxy-1,3-benzodioxol-5-yl)-2-propen-1-ol: no relevant information.	4
C_9_H_19_NO_4_	Panthenol	Is the alcohol equivalent of pantothenic acid (vitamin B5), an essential nutrient necessary for the synthesis of coenzyme A, which plays an important role in epithelial protein metabolism [28]. Topical application promotes skin regeneration and wound healing [29].	2
C_6_H_6_O_2_	Resorcinol	Not a naturally occurring metabolite exerting keratolytic activity often found in topical pharmaceutical products as an antiseptic [30,31].	2

## Data Availability

The dataset is not made publicly available due to privacy restrictions.

## Supplementary Materials

The following supporting information can be downloaded at: https://www.mdpi.com/article/10.3390/metabo13111125/s1, Table S1: The global metabolomic analyses.
