# Supplementary material for: Tear and Saliva Metabolomics in Evaporative Dry Eye Disease in Females
Source: Metabolites. 2023 Nov 2;13(11):1125. doi: 10.3390/metabo13111125 (PMC10673540; doi:10.3390/metabo13111125)
Supplement: Supplementary file 1 [file metabolites-13-01125-s001.zip › metabolites-2664298-Supplementary.pdf]

Positive ionization - Increased in patients

Name	Formula	Molecular Weight	RT [min]	Ratio: (Patient) / (Control)	P-value: (Patient) / (Control)	Adj. P-value: (Patient) / (Control)	P_value controls<br>Shapiro-Wilk	P_value patients<br>Shapiro-Wilk	Mann-Whitney U test
Spermine	C10 H26 N4	202.21572	1.426	2.292	0.036183746	0.1334355	0.004	0.009	.025[b]
Spermidine	C7 H19 N3	145.15788	1.489	2.205	0.002922715	0.040120087	0.039	0.296	.003[b]
	C4 H12 N2 O2	120.08982	2.264	39.672	0.001791744	0.031385379	<.001	0.024	.003[b]
	C6 H16 N2 O3	164.11602	2.294	36.528	8.76099E-05	0.010649572	<.001	0.901	<.001[b]
Triethanolamine	C6 H15 N O3	149.10513	2.307	3.929	0.038607381	0.137621729	0.002	0.005	.059[b]
	C5 H13 N O2	119.0946	2.316	2.809	0.002823107	0.040120087	0.011	0.479	.007[b]
	C8 H19 N O4	193.13135	2.32	64.928	0.000517755	0.015115568	<.001	0.908	<.001[b]
	C7 H17 N O3	163.12077	2.357	5.851	0.000340984	0.01185302	<.001	0.86	<.001[b]
	C2 H2 N4 O2 S	145.98914	2.669	2.119	0.018090612	0.092778509	0.215	<.001	.002[b]
	C10 H23 N O5	237.15753	2.698	60.779	0.000114486	0.010649572	<.001	<.001	<.001[b]
	C9 H19 N O4	205.13134	2.75	327.4	0.000328932	0.01185302	<.001	0.686	<.001[b]
	C8 H19 N O3	177.13641	2.87	22.97	0.00091706	0.019669992	<.001	0.184	<.001[b]
D-(+)-Carnitine	C7 H15 N O3	161.10509	2.954	18.566	0.006796324	0.053877179	<.001	0.126	.020[b]
O-(2-aminoethyl)serine	C5 H12 N2 O3	148.08471	3.399	13.346	0.002002042	0.032877277	<.001	0.45	.002[b]
	C11 H8 S	172.03471	3.427	6.295	0.006046151	0.051909263	0.009	0.855	.008[b]
	C11 H23 N O5	249.15755	3.836	261.345	0.000171795	0.010649572	<.001	0.531	<.001[b]
Diethylene glycol	C4 H10 O3	106.06295	4.12	3.689	0.000784192	0.019167104	0.005	0.232	.001[b]
3-hydroxybutyric acid	C4 H8 O3	104.04735	5.82	2.173	0.038729096	0.137621729	0.076	0.046	.059[b]
(2S)-2-Piperazinecarboxylic acid	C5 H10 N2 O2	130.07424	7.408	15.992	0.00293934	0.040120087	<.001	0.074	.002[b]
	C7 H11 N2 O5 P	234.04154	8.005	2.083	0.001993033	0.032877277	0.101	0.54	.001[b]
Daminozide	C6 H12 N2 O3	160.08468	8.224	10.688	0.002284889	0.036944896	<.001	0.568	.004[b]
	C14 H11 F N2 O	242.08781	10.624	39.144	0.000148942	0.010649572	<.001	0.691	<.001[b]
	C5 H8 N8 O	196.08232	11.53	6.015	0.003858806	0.044567083	<.001	0.746	.003[b]
	C32 H59 N2 O4 P3	628.36901	11.916	3.855	0.031244789	0.122350252	<.001	0.008	.035[b]
	C11 H4 N10	276.0609	12.676	5.103	0.000896	0.019618671	0.004	<.001	.002[b]
Glycyltyrosine	C11 H14 N2 O4	238.09536	12.945	301.056	0.000226078	0.011026434	<.001	0.003	<.001[b]
		181.09909	13.119	2.142	0.046786694	0.15132672	0.069	0.505	.035[b]
	C11 H14 N2 O4	238.09518	13.153	94.455	0.000170913	0.010649572	<.001	0.64	<.001[b]
	C24 H20 O7	420.12056	13.684	7.111	0.006152765	0.051909263	0.01	0.89	.008[b]
Dacarbazine	C6 H10 N6 O	182.09174	13.759	2.711	0.015934596	0.084873119	<.001	0.001	.053[b]
	C11 H20 N2 O	196.15751	13.905	2.197	0.038760437	0.137621729	0.14	0.549	.010[b]
12-Hydroxylauric acid	C12 H24 O3	216.17237	14.277	2.948	0.011884076	0.071782554	0.002	0.77	.016[b]
	C18 H21 N4 O6 P	420.12056	14.477	11.377	0.008662627	0.063937003	0.007	0.59	.011[b]
	C17 H18 N2 O4	314.12633	14.49	29.326	0.049468962	0.151579693	<.001	0.016	.101[b]
	C16 H24 O8	366.12864	14.717	2.574	0.010170171	0.067900068	0.199	0.033	.003[b]
	C12 H20 N6 O3	296.15979	15.136	3.602	0.006223185	0.051909263	0.005	0.016	<.001[b]
								n =	32

Positive ionization - Decreased in patients

Name	Formula	Molecular Weight	RT [min]	Ratio: (Pasient) / (Kontroll)	P-value: (Pasient) / (Kontroll)	Adj. P-value: (Pasient) / (Kontroll)	Shapiro-wilk	Shapiro-wilk	
ETHANOLAMINE	C2 H7 N O	61.053	2.19	0.34	0.000690966	0.018954504	0.098	0.422	<.001[b]
Triethanolamine	C5 H11 N O4	149.06873	2.291	0.266	0.003703007	0.044309369	0.025	0.049	<.001[b]
Bicine	C6 H13 N O4	163.08441	2.296	0.133	0.004993746	0.049651358	0.472	<.001	.001[b]
Diethanolamine	C4 H11 N O2	105.07897	2.405	0.124	0.004555109	0.047874198	0.11	<.001	.007[b]
Pyridoxal	C8 H9 N O3	167.0582	3.255	0.389	0.001016565	0.01978537	0.76	<.001	<.001[b]
Inosine	C10 H12 N4 O5	268.08075	7.027	0.383	0.04775011	0.15132672	0.33	0.017	.078[b]
D-Panthenol	C9 H19 N O4	205.13136	10.625	0.044	0.015164293	0.082943236	0.003	<.001	.012[b]
1,2-Cyclohexanediol	C6 H12 O2	116.0837	12.319	0.038	0.027617181	0.114658842	0.028	<.001	.101[b]
	C8 H15 N2 O5 P	250.07275	12.323	0.043	0.044073415	0.146123529	0.022	<.001	.306[b]
5-Hydroxydecanoic acid	C10 H20 O3	188.14117	13.035	0.114	0.038423239	0.137621729	0.001	<.001	.191[b]
	C10 H22 O4	206.15178	13.046	0.034	0.042838894	0.144107073	0.002	<.001	.093[b]
	C7 H14 N3 O4 P	235.07321	13.21	0.249	0.005524259	0.050486926	0.089	0.05	.001[b]
	C5 H11 N6 O3 P	234.06404	13.21	0.49	0.003752173	0.044309369	0.093	0.002	.001[b]
Hydroquinone	C6 H6 O2	110.03679	13.706	0.104	0.012999939	0.074660853	0.054	<.001	.039[b]
Oleamide	C18 H35 N O	281.2717	19.06	0.226	0.000318803	0.01185302	0.064	<.001	<.001[b]
Hydroxydocosanoic acid	C22 H44 O3	356.32878	21.385	0.399	0.004787016	0.04932504	0.859	0.049	.007[b]
								n =	11

Negative ionization - Increased in patients

Name	Formula	Molecular Weight	RT [min]	Ratio: (Pasient) / (Kontroll)	P-value: (Pasient) / (Kontroll)	Adj. P-value: (Pasient) / (Kontroll)	Shapiro-wilk	Shapiro-wilk	
	C5 H13 N5 P2	205.0638	2.396	3.972	0.023510477	0.103299694	0.045	0.009	.001[b]
3-(3-Hydroxy-4-methoxyphenyl)-2-oxiranecarboxylic ac	C10 H10 O5	210.05307	12.631	4.263	0.001686027	0.103299694	0.014	0.009	.001[b]
Silandrin	C25 H22 O9	466.12682	13.721	7.695	0.005644712	0.103299694	0.011	0.565	.008[b]
Arachidonic acid	C20 H32 O2	304.24057	20.442	2.102	0.027967034	0.103299694	0.057	0.015	.039[b]
Retinyl acetate	C22 H32 O2	328.24064	21.014	5.128	0.012091755	0.103299694	0.018	0.001	.012[b]
								n =	5

Negative ionization - Decreased in patients

Name	Formula	Molecular Weight	RT [min]	Ratio: (Pasient) / (Kontroll)	P-value: (Pasient) / (Kontroll)	Adj. P-value: (Pasient) / (Kontroll)	Shapiro-wilk	Shapiro-wilk	
2-(alpha-D-mannosyl)-D-glyceric acid	C9 H16 O9	268.07942	2.808	0.018	0.008603229	0.103299694	<.001	<.001	.152[b]
Panthenol	C9 H19 N O4	205.13163	10.603	0.053	0.021710298	0.103299694	0.003	<.001	.020[b]
3,4,5-trimethoxycinnamic acid	C12 H14 O5	238.08436	13.183	0.007	0.003354868	0.103299694	0.012	<.001	.003[b]
Resorcinol	C6 H6 O2	110.03682	13.704	0.091	0.008970187	0.103299694	0.047	<.001	.028[b]
	C16 H14 O6	302.07937	13.729	0.458	0.009411666	0.103299694	0.01	<.001	.008[b]
Hydroxydocosanoic acid	C22 H44 O3	356.32952	21.381	0.424	0.006690856	0.103299694	0.407	0.04	.011[b]
Adipid adic di(2-ethylhexyl) ester	C22 H42 O4	370.30889	21.415	0.456	0.01157665	0.103299694	0.466	0.018	.011[b]
								n =	6

Saliva

Name	Formula	Molecular Weight	RT [min]	Ratio: (Pasient) / (Kontroll)	P-value: (Pasient) / (Kontroll)	Adj. P-value: (Pasient) / (Kontroll)	Shapiro-wilk	Shapiro-wilk	
Hypotaurine	C2 H7 N O2 S	109.01979	2.276	0.36	0.009773547	0.659960878	0.122	0.012	.015[b]
Fluorescein	C20 H12 O5	332.0681	14.106	11.822	9.19982E-06	0.00624668	0.52	<0.001	.003[b]
									2
								Total significant=	56
